# Treating type 2 diabetes in COVID-19 patients: the potential benefits of injective therapies

**DOI:** 10.1186/s12933-020-01090-9

**Published:** 2020-07-22

**Authors:** Miriam Longo, Paola Caruso, Maria Ida Maiorino, Giuseppe Bellastella, Dario Giugliano, Katherine Esposito

**Affiliations:** 1 Division of Endocrinology and Metabolic Diseases, Department of Advanced Medical and Surgical Sciences, University of Campania “Luigi Vanvitelli”, Piazza Luigi Miraglia 2, 80138 Naples, Italy; 2 Diabetes Unit, Department of Advanced Medical and Surgical Sciences, University of Campania “Luigi Vanvitelli”, Naples, Italy

**Keywords:** Type 2 diabetes, Hyperglycemia, COVID-19, Cytokine storm, GLP-1RAs, Insulin

## Abstract

The coronavirus disease 2019 (COVID-19) has been declared as pandemic by the World Health Organization and is causing substantial morbidity and mortality all over the world. Type 2 diabetes, hypertension, and cardiovascular disease significantly increase the risk for hospitalization and death in COVID-19 patients. Hypoglycemia and hyperglycemia are both predictors for adverse outcomes in hospitalized patients. An optimized glycemic control should be pursued in patients with diabetes and SARS-CoV-2 infection in order to reduce the risk of severe COVID-19 course. Both insulin and GLP-1RAs have shown optimal glucose-lowering and anti-inflammatory effects in type 2 diabetic patients and may represent a valid therapeutic option to treat asymptomatic and non-critically ill COVID-19 diabetic patients.

## Introduction

On March 2020, the World Health Organization (WHO) declared the pandemic caused by severe acute respiratory syndrome coronavirus 2 (SARS-CoV-2) and officially named the disease “coronavirus disease 2019” (COVID-19). On May the 19th 2020, the disease has rapidly spread to more than 200 countries with 316,289 deaths and 4,735,622 confirmed cases in the globe. However, these numbers may be even higher, as many COVID-19 cases have been unidentified and unreported in most countries, especially in those with lower health standards measured by the Healthcare Access and Quality Indices (HAQ Index) [1].

The spectrum of the disease is highly heterogeneous: from the lack of symptoms or mild fever to the need of hospital admission in intensive care unit (ICU) for pneumonia, sepsis, respiratory failure, and acute respiratory distress syndrome (ARDS). There is accumulating evidence that ARDS and respiratory failure by COVID-19 may be caused by a defective immune response, characterized by a rapid proliferation and hyperactivation of T cells, macrophages, natural killer cells, and an overproduction of more than 150 chemical mediators (the so called “cytokines storm”), including pro-inflammatory cytokines (TNF-α, INF-γ, IL-6, IL-1β, IL-8), and chemokines (CCL-2, CCL-3, CCL-5, CXCL-8, CXCL-10), leading to an increased vascular permeability and multiple-organ failure [2, 3]. Furthermore, high levels of IL-6 may have negative impact on cardiovascular system, promoting cardiomyopathy and myocardial dysfunctions. In addition, the cytokine storm is responsible for the impairment of the endothelial function, which can result in capillary leakage, hypotension, and coagulopathy, responsible for a more severe clinical course of COVID-19 [3]. Recently, a promising therapeutic strategy with a monoclonal antibody inhibitor of IL-6 receptor (tocilizumab) is currently under evaluation in clinical trials for the treatment of COVID-19 pneumonia. Moreover, SARS-CoV-2 is associated with increased risk of acute cardiovascular events, including myocardial infarction, myocarditis, heart failure, arrhythmias, venous thromboembolic events and renal failure [4]. Therefore, full attention should be paid to the prevention and treatment of comorbidities and cardiovascular risk factors.

## Diabetes as a risk factor for worse COVID-19 outcomes

Type 2 diabetes, hypertension, and cardiovascular diseases have been identified as the most common comorbidities for SARS-CoV-2 infection and have been associated with worse outcomes and more severe course of COVID-19 [5]. Moreover, about 30% of diabetic people present with a concomitant cardiovascular disease (CVD), further weakening the clinical status of individuals who are per se susceptible to the viral infection. Almost one third of patients deceased by COVID-19 was affected by diabetes mellitus in recent studies from China and Unites States [5, 6]; patients with diabetes have a twofold increase in fatal outcomes than those without [7]. Specifically, people with diabetes were more prone to invasive mechanical ventilation, admission in the ICU and development of acute kidney injury, as compared with patients without diabetes [6]. Possible explanations for the major severity of COVID-19 in diabetes include increased susceptibility to infections, dysregulation of innate immune response and defects of cell-mediated immunity [8]. Type 2 diabetes is characterized by a status of low-grade chronic inflammation, expressed by increased levels of mediators of flogosis including TNF-α, C-reactive protein (CRP), IL-1, IL-6, leptin, resistin [8]. Moreover, type 2 diabetes is associated with increased oxidative stress, platelet aggregation and endothelial dysfunction. Globally, all these alterations may represent the underlying conditions linking diabetes to other chronic pathologies, including hypertension and cardiovascular diseases. Acute hyperglycemia during infection may further contribute to a huge increase in inflammatory mediators, which could in turn enhance the risk of multiple-organ failure and acute cardiovascular event [9]. Moreover, the severe systemic inflammation and the status of hypercoagulability enhances the risk of atherosclerotic plaque disruption and acute myocardial infarction (AMI) in patients with COVID-19 [4]. However, the suggested approach to treat patients with AMI in the context of SARS-CoV-2 pandemic is primary percutaneous coronary intervention (PCI) for patients with an ST elevation myocardial infarction (STEMI) at PCI capable hospitals when it can be provided in short time; on the other hand, a fibrinolysis-based therapy should be performed at non-PCI capable referral hospitals or in specific situations where primary PCI cannot be executed or is not deemed the best option [10].

## Diabetes injective treatments: a potential aid against COVID-19?

Hypoglycemia and hyperglycemia are both predictors for adverse outcomes in hospitalized patients. An optimized glycemic control should be pursued in patients with diabetes and SARS-CoV-2 infection, avoiding hyperglycemic peaks and hypoglycemic events, and blunting inflammatory status in order to reduce the risk of severe complications. The preferred therapeutic options for glucose control with low glucose variability in non-critically ill COVID-19 patients may comprise subcutaneous insulin and GLP-1 receptor agonists (GLP-1RAs).

### Insulin

Insulin associated with a constant glucose monitoring is the first-choice treatment for hyperglycemia in hospital settings. An intensive regimen with basal and prandial insulin analogues is the best treatment for non-critically ill hospitalized patients with good or poor nutritional oral intake, in order to reach the recommended target glucose range of 140–180 mg/dL.

In an experimental model of influenza, insulin has shown to exert immuno-stimulatory effects on T cells by activating insulin receptor-intracellular cascade, strengthening the host defense against the infection. In a study on 451 hospitalized patients with critical illness needing prolonged intensive care at ICU, intensive insulin therapy was associated with reduced levels of inflammatory markers (mannose-binding lectin and CRP), as compared with conventional treatment. Moreover, in a study of 1200 patients admitted at ICU, of whom 203 were affected by diabetes, intensive insulin therapy significantly reduced the rate of morbidities, including newly acquired kidney injury and the need of mechanical ventilation, with accelerated discharge from the ICU and the hospital. There is still paucity of evidence about the potential benefits or risks associated with the use of intensive insulin regimen in people affected by COVID-19 with or without diabetes. However, keeping in mind that the preferred option for critically ill patients is based on intensive intravenous insulin regimen, using insulin with a perfusion device should be suggested for diabetic patients affected by COVID-19 treated in the ICU setting [11].

### GLP-1RAs

GLP-1RAs are effective glucose-lowering injective drugs used in the treatment of type 2 diabetes. Physiologically, GLP-1 release is typically increased in response to: (1) enteral nutrients to stimulate insulin secretion and lower glucose levels; (2) inflammatory stimuli (i.e. endotoxin, IL-1β, and IL-6) in order to attenuate inflammatory response. Higher levels of circulating GLP-1 are found in patients with sepsis and correlate with its severity and mortality [12]. In studies of cultured macrophages, treatment with exendin-4 reduced levels of inflammatory cytokines, as IL-1β, TNF-α, IL-6 and IFN-γ. Moreover, in vitro studies on human umbilical vein endothelial cells showed that liraglutide reduced reactive oxygen species and the vasoconstrictor peptide endothelin-1, improving endothelial function and peripheral vasodilatation. These anti-inflammatory effects have been confirmed in patients with type 2 diabetes and obesity. Administration of exenatide lowered the expression of genes coding for TNFα, IL-1β, JNK-1 and decreased IL-6 concentrations in 24 obese patients with type 2 diabetes [12]. Moreover, intravenous infusions of exogenous GLP-1, exenatide and liraglutide were associated with the improvement of blood glucose levels, with less risk of hypoglycemia and lower insulin doses in several small clinical trials conducted in peri-operative and critical care settings at ICU [12]. In a multicenter open-label randomized controlled trial, exenatide alone or in combination with basal insulin showed to be safe and effective for the management of hyperglycemia when compared with basal-bolus insulin regimen in 150 hospitalized general medical and surgical patients with type 2 diabetes. Furthermore, GLP-1RAs have been associated with prevention of cardiovascular and kidney disease in diabetic population, which is ever more important in diabetic patients affected by SARS-CoV-2 [13]. In a 26 weeks double-blind trial on 23 type 2 diabetic patients without prior cardiovascular events, liraglutide reduced left ventricular filling pressure, thereby lowering the risk of heart failure with preserved ejection fraction [14]. Moreover, left ventricular systolic function parameters (stroke volume and ejection fraction) reduced to normal range.

The results of cardiovascular outcome trials (CVOTs) in people with type 2 diabetes mellitus and increased cardiovascular risk have demonstrated a significant benefit in terms of reduction in time to first major adverse cardiovascular event (MACE) with the use of liraglutide, semaglutide, albiglutide and dulaglutide [15]. Specifically, dulaglutide showed to be beneficial even in a large proportion of type 2 diabetic patients without CVD in the REWIND study. In a post hoc analysis of SUSTAIN 6, including 3297 subjects with type 2 diabetes and high CV risk, once-weekly semaglutide reduced the risk of MACE and each MACE component in all subjects included in the trial, regardless of gender, age, or baseline CV risk profile, as compared with placebo [16]. Finally, In a recent meta-analysis on 56,004 patients from 7 CVOTs (68.9% patients with established cardiovascular disease), GLP-1RAs reduced the risk of MACE by 13%, of cardiovascular death by 12%, of all-cause mortality by 11%, of non-fatal stroke by 16%, of hospitalization for heart failure by 9%, of macroalbuminuria by 24% [13].

These characteristics may render GLP-1RAs an attractive therapeutic option to treat asymptomatic and non-critically ill COVID-19 patients with type 2 diabetes, in which acute hyperglycemia and cytokine storm could worse the prognosis. Of note, in the recent consensus report by the American Diabetes Association (ADA) and the European Association for the Study of Diabetes (EASD), the use of GLP-1RAs with proven cardiovascular benefits should be preferred for patients with type 2 diabetes who have established atherosclerotic cardiovascular disease or indicators of high risk. Data on GLP-1RAs use in patients with COVID-19 disease are actually scanty. All COVID-19 diabetic patients on GLP-1RA treatment should be closely monitored and provided with regular meals and adequate fluid intake to prevent the risk of dehydration [17].

### Combination of basal insulin and GLP-1RAs

**Fig. 1:**
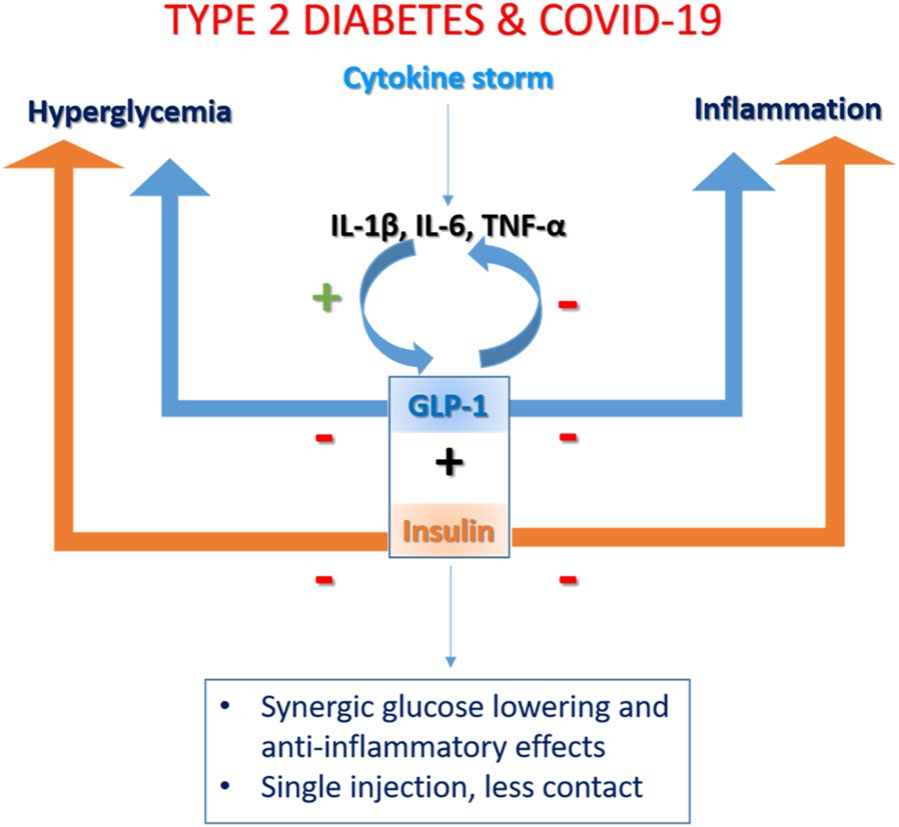
Type 2 diabetes and COVID-19 share a common soil of chronic inflammation. High levels of pro-inflammatory cytokines IL-1β, IL-6, TNF-α stimulate secretion of GLP-1, which in turn is able to reduce their circulating levels, hyperglycemia and inflammation. Similarly, insulin lowers both glucose levels and inflammation. The combination of GLP-1 and insulin provides synergic glucose lowering and anti-inflammatory effects, with the benefit of a single injection and less frequency of contacts. +: stimulation; −: inhibition

The association of GLP-1RA and basal insulin, already available as free or fixed-ratio combo, may be another therapeutic strategy to treat asymptomatic and non-critically ill COVID-19 patients with type 2 diabetes. This combination therapy provides a similar glycemic control as compared with intensive insulin regimen, associated with lower risk of hypoglycemia and weight gain [17]. Combining these drugs may have a rationale in the treatment of diabetes outside the critical care of COVID-19 thanks to the synergic glucose lowering effect and the potential anti-inflammatory actions of both GLP-1 and insulin (Fig. 1). Moreover, the administration of the two drugs with a single daily injection avoids unnecessary exposure to COVID-19 patients, minimizing the risk of infection transmission. In summary, both GLP-1RA and insulin, alone or in combination, may be effective therapeutic options for asymptomatic and non-critically ill COVID-19 diabetic patients.

## Conclusions

COVID-19 is a rapidly spreading pandemic. Type 2 diabetes is one of the principal comorbidity of patients with COVID-19, resulting as an independent predictor for worse outcomes. Maintaining a good glycemic control is mandatory, as both hyperglycemia and hypoglycemia are associated with enhanced inflammatory profile and acute cardiovascular event. Given the effective glucose-lowering and anti-inflammatory effects, both insulin and GLP-1RAs, alone or in combination, may represent a valid therapeutic option to treat asymptomatic and non-critically ill COVID-19 diabetic patients. However, more prospective studies are needed to evaluate whether the use of these glucose-lowering injective agents correlate with better outcomes and lower morbidity and mortality in the context of SARS-CoV-2.

## Data Availability

Not applicable.

## Abbreviations

ADA: American Diabetes Association
ARDS: Acute respiratory distress syndrome
COVID-19: Coronavirus disease 2019
CRP: C-reactive protein
CVD: Cardiovascular disease
CVOTs: Cardiovascular outcome trials
EASD: European Association for the Study of Diabetes
ICU: Intensive care unit
GLP-1RAs: Glucagon-like peptide-1 receptor agonists
MACE: Major adverse cardiovascular event
REWIND: Researching Cardiovascular Events with a Weekly Incretin in Diabetes Trial
SARS-CoV-2: Severe acute respiratory syndrome coronavirus 2
WHO: World Health Organization
